# Mucosa-associated lymphoid tissue (MALT) lymphoma as an unusual cause of malignant hilar biliary stricture: a case report with literature review

**DOI:** 10.1186/s12957-016-0928-z

**Published:** 2016-06-24

**Authors:** Yong Keun Park, Jee Eun Choi, Woon Yong Jung, Sung Kyu Song, Jong In Lee, Chul-Woon Chung

**Affiliations:** Department of Surgery, Catholic Kwandong University International St. Mary’s Hospital, Incheon, South Korea; Catholic Kwandong University College of Medicine, Incheon, South Korea; Department of Pathology, Catholic Kwandong University International St. Mary’s Hospital, Incheon, South Korea

**Keywords:** B cell lymphoma, Hilar cholangiocarcinoma, Klatskin tumor, Magnetic resonance cholangio-pancreatography

## Abstract

**Background:**

Biliary strictures at the hilum of the liver arise from heterogeneous etiologies. The majority is malignant entities, but some may have benign etiologies. It is difficult to distinguish between malignant and benign biliary strictures preoperatively. It has been reported that 5~15 % of preoperative diagnoses of hilar cholangiocarcinoma turn out to be benign lesions or even other types of malignancies. Primary non-Hodgkin’s lymphoma of the extrahepatic bile duct is very rare, with only a few cases reported as mucosa-associated lymphoid tissue (MALT) lymphoma arising from the hepatic duct bifurcation. We herein report a case of a female patient presenting with perihilar bile ducts obstructed by primary MALT lymphoma resembling hilar cholangiocarcinoma, along with a review of the literature.

**Case presentation:**

An 86-year-old female was referred to our hospital manifesting obstructive jaundice and abdominal pain. The reported imaging studies revealed distended intrahepatic bile duct with the stricture of common hepatic duct including bifurcation, which was suspicious of cholangiocarcinoma of the bile duct. The initial laboratory-confirmed cholestasis with a total bilirubin of 8.6 mg/dL, aspartate amino transferase (AST) 178 U/L, alanine transferase (ALT) 105 U/L, and the tumor marker CA 19-9 was elevated with a value of 167 U/mL. Viral markers for hepatitis B and C viruses were negative. She underwent extrahepatic bile duct resection and hepaticojejunostomy. Histological examination of the resected specimen revealed MALT lymphoma. Postoperative follow-up of 1 year has been completely uneventful, without any symptoms or disease recurrence.

**Conclusions:**

In exceptional cases, in which radiologic and clinical features point to cholangiocarcinoma, the actual reason for obstructive jaundice and abdominal pain can be a non-Hodgkin’s lymphoma. In the case of a MALT lymphoma, it can be cured with complete resection.

## Background

Space-occupying lesions at any level of the biliary tree impede bile flow and present as diffuse or focal dilatation of upstream bile ducts on radiographic studies. In correlation with clinical and biochemical features, intrahepatic bile ducts with diameters exceeding 40 % of that of adjacent portal veins and extrahepatic bile ducts with diameters greater than 7 mm on ultrasound and 10 mm on computed tomography (CT) both imply a narrowing of the bile duct. As such, further evaluation of presumed biliary stricture is required to determine location and extent. The majority of biliary strictures are malignant, but up to 30 % may arise from benign etiologies [1]. There is no precise method of preoperatively distinguishing malignant from benign biliary strictures, which means that up to 20 % of patients undergoing surgical resection for suspected biliary malignancies actually have a benign disease [2, 3].

Hilar cholangiocarcinoma, also known as Klatskin tumor, is a malignant tumor that narrows the confluence of the left and right hepatic ducts. Only 20–30 % of patients are potentially curable with surgical resection, a major operation with a less than promising oncologic outcome [4]. As such, some physicians are trying to avoid the surgical option, as it may actually worsen the clinical condition. Histopathological studies reveal 5–15 % of these to be benign lesions or other malignancies that are curable by complete resection [5].

Primary low-grade B cell lymphoma of mucosa-associated lymphoid tissue (MALT) is a subtype of non-Hodgkin’s lymphoma. Thirty-four cases of primary non-Hodgkin’s lymphoma of the extrahepatic bile duct have been reported, while only four cases of MALT lymphoma arising from the hepatic duct bifurcation have. We elaborate here on the case of an 86-year-old female patient, who presented with perihilar bile duct obstruction by primary MALT lymphoma resembling hilar cholangiocarcinoma.

## Case presentation

**Fig. 1:**
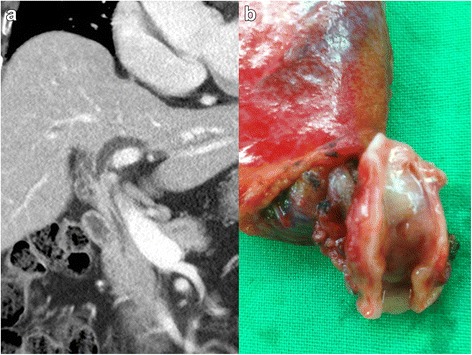
Preoperative computed tomographic (**a**) and gross (**b**) findings. **a** Diffuse dilatation of the intrahepatic duct and wall thickening of the common bile duct were shown. **b** Wall thickening with focal mass was found without complete obstruction by mass

**Fig. 2:**
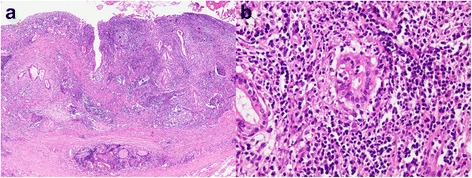
Microscopic findings of resected tumor. **a** The common bile duct showed diffuse infiltration of lymphoid cells forming lymphoid follicles. The glands at the mucosal surface were extensively eroded by lymphocytic infiltration (hematoxylin-eosin (HE) ×40). **b** The neoplastic lymphocytes occasionally infiltrated into the glandular epithelium (lymphoepithelial lesion; HE ×400)

**Fig. 3:**
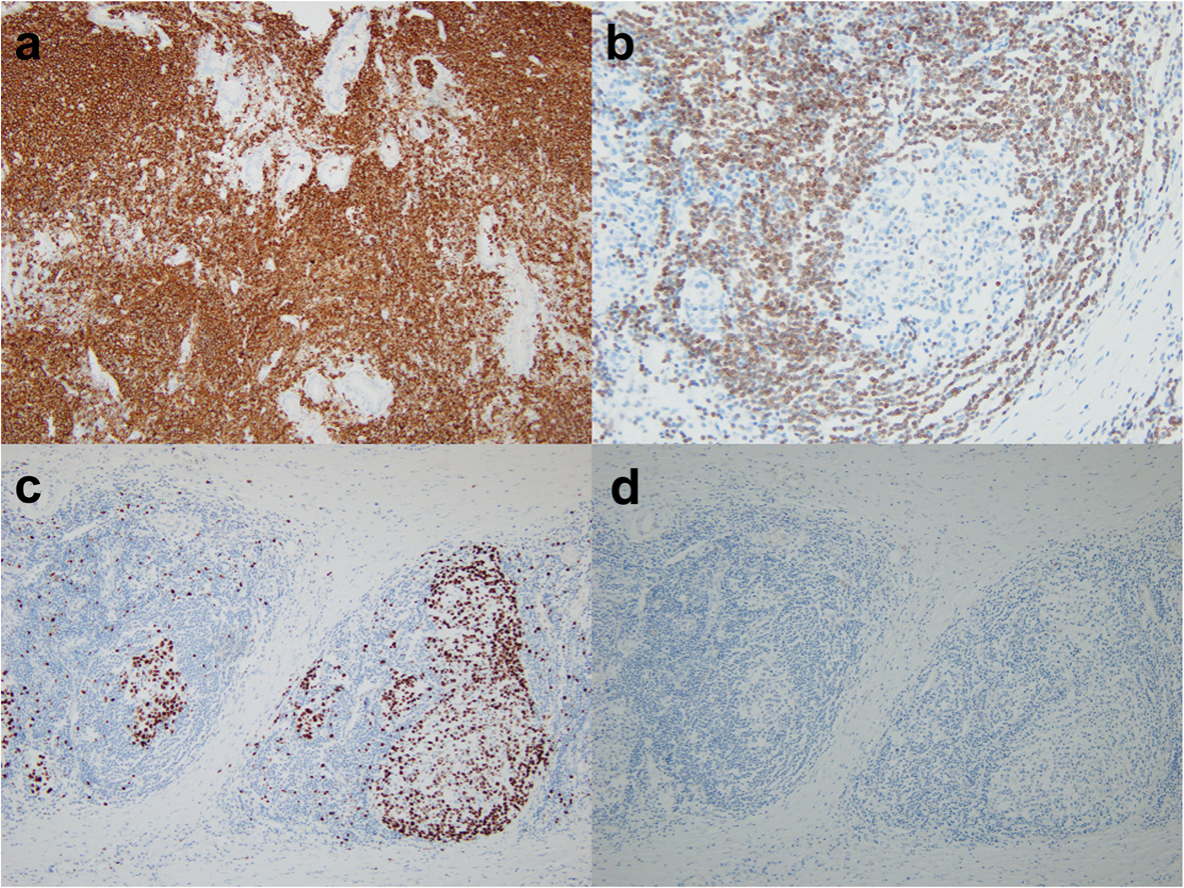
Immunohistochemical staining. **a** CD20 immunohistochemical stain revealed diffuse infiltration of B cells (CD20 ×100). **b** BCL2-positive neoplastic cells surrounded reactive germinal centers containing proliferating B cells (BCL2 ×200) (**c**), which were highlighted by Ki-67 (Ki-67 ×100). **d** Immunohistochemical staining results for cyclin D1 protein were negative (cyclin D1 ×100)

An 86-year-old female was referred to our hospital for treatment of a hilar biliary stricture lesion on abdominal CT initially performed to evaluate jaundice and vague abdominal pain. She complained of yellow skin and eyes that began 2 weeks prior. Around the same time, she also began experiencing abdominal pain that was dull and intermittent. The pain did not radiate nor was it related to meals; it was sometimes accompanied by anorexia and nausea. She was constipated but reported no changes in the color of stool. The patient also did not notice weight changes. She had a past medical history of diabetes mellitus controlled by metformin for the past 2 years and had no history of surgery. Her family history was non-contributory. Initial physical examination revealed no specific findings. Contrast-enhanced CT showed a distended intrahepatic bile duct with a stricture at the common hepatic duct that included the bifurcation (Fig. 1a). This finding was suspicious for cholangiocarcinoma of the bile duct. Direct invasion of nearby vascular structures and distant metastases were not detected. The initial laboratory data suggested cholestasis, with a total bilirubin of 8.6 mg/dL, aspartate amino transferase 178 U/L, and alanine transferase 105 U/L. Furthermore, the level of carbohydrate antigen 19-9 as a tumor marker was elevated at 167 U/mL. The viral markers were HBsAg (−), HBsAb (−), and anti-HCV (−). She was negative for HIV. As the patient had fever and leukocytosis, intravenous antibiotics were started out of suspicion for concurrent cholangitis. Immediately after administration of antibiotics, the bilirubin level dramatically decreased and was within normal range a week later. The lesion was not thought to have completely blocked biliary outflow. Preoperative evaluation of the patient’s cardiac and pulmonary function was unremarkable, with echocardiogram showing no definite cardiac dysfunction. The patient’s hemoglobin and hematocrit levels were mildly decreased to 11.7 g/dL and 34.2 %, respectively. The serum blood urea nitrogen and creatinine were 8.5 and 0.6 mg/dL, respectively. There was no serum electrolyte imbalance or coagulopathy found. Her performance status was appropriate. Due to suspected hilar cholangiocarcinoma, extrahepatic bile duct resection and biliary reconstruction (Roux-en-Y hepaticojejunostomy) were planned. After careful exploration of the peritoneal cavity, hepatoduodenal dissection was performed, following cholecystectomy. On manual examination of the common bile duct (CBD), a mass was palpated. However, this mass was soft and had not invaded any neighboring tissue, including the hepatic artery and portal vein. The portion of the CBD containing the mass was easily separated from the surrounding hepatoduodenal connective tissues. The proximal resection margin was the nearby bifurcation of the hepatic duct, and the distal margin was just above the intra-pancreatic portion (Fig. 1b). The light microscopic examination revealed diffuse infiltration of atypical lymphocytes in the common bile duct. They formed lymphoid follicles and occasionally infiltrated into glandular epithelium resulting in the so-called lymphoepithelial lesion (Fig. 2). On immunohistochemical staining, CD20 was diffusely positive in neoplastic cells and CD3 was expressed in scattered T cells. The neoplastic cells were BCL2 positive but germinal center B cells were negative. Also, the neoplastic cells were negative for cyclin D1 (Fig. 3). The final diagnosis was extranodal marginal zone lymphoma of MALT lymphoma arising in the common bile duct. Postoperatively, the patient had no serious complications. By postoperative day 2, she was tolerating a liquid diet. She was discharged on the 12th postoperative day. No additional chemotherapy was considered, as complete resection of the tumor had been achieved. At 1-year post-operation, her recovery had been completely uneventful, without recurrence of any symptoms of disease.

### Discussion

An extranodal marginal zone B cell lymphoma, also called low-grade B cell lymphoma of MALT, arises in a number of epithelial tissues, including the stomach, salivary gland, lung, small intestine, and thyroid. It has a tendency to remain localized to the tissue of origin over time, but does recur frequently, with potential for systemic spread and transformation to a high-grade B cell lymphoma. The pathogenesis of MALT lymphoma owes to chronic inflammation of the tissue, which leads to the local accumulation and proliferation of antigen-dependent B cells and T cells. With time, B cell clones emerge, still dependent on antigens for growth and survival. At the stage of monoclonal proliferation, these cells are not able to spread beyond the site of inflammation. With acquisition of additional mutations, however, the tumor becomes antigen-independent and capable of systemic spread [6]. This patient had no known history of prior cholangitis or any evidence of hepatitis, so it is hard to be suspicious of any chronic antigenic stimulation which carries etiologic specificity. The underlying etiologic mechanism of bile duct involvement by this type of lymphoma should be further elucidated.

Primary non-Hodgkin’s lymphoma of the extrahepatic bile duct presenting as obstructive jaundice is extremely rare, with lymphoma occupying the perihilar bile duct being even more uncommon. In 2005, the first case was reported of primary MALT lymphoma arising from the perihilar bile ducts. Primary MALT lymphoma is such a rare finding at this site that only four cases have been reported thus far, making it difficult to suspect MALT lymphoma in such cases [7]. It is challenging to differentiate MALT lymphoma of the perihilar bile ducts from hilar cholangiocarcinoma, the most common form of bile duct cancer. The two types of malignancies are indistinguishable both clinically and radiologically. We reviewed three cases of patients whose diagnoses could not be confirmed as primary MALT lymphoma until radical resection of the bile duct was performed; we further included one patient who had been confirmed by biopsy and had undergone chemotherapy [7–10] (Table 1).

**Table 1:** Summary of cases of primary mucosa-associated lymphoid tissue lymphoma resembling Klatskin tumor

Case	Author, year	Age/sex	Radiologic findings	Surgical findings	Treatment
1	Shimura et al. [7]	59/male	Irregular, incomplete stenosis from hilum to the lower part of the bile duct	Mass (4.5 × 3.5 cm) around the EHD	Right trisegmentectomy + caudate lobectomy
2	Shito et al. [9]	71/male	Dilatation of IHD with a circumscribed heterogeneous mass in the main hepatic junction	Dense, white, nodular mass (5.0 × 2.5 cm) in the main hepatic junction	Left hemihepatectomy + caudate lobectomy + radical bile duct resection + Roux-en-Y hepaticojejunostomy + chemotherapy (CHOP, 3 cycles)
3	Yoon et al. [8]	62/male	Dilatation of IHD with long, segmental, circumferential wall thickening of entire extrapancreatic portion of CBD + cystic duct + right posterior, anterior segmental IHD + left secondary biliary confluence	Diffuse wall thickening of EHD with smooth inner and outer surface without mass lesion	Right hemihepatectomy + caudate lobectomy + radical bile duct resection + Roux-en-Y hepaticojejunostomy + chemotherapy (CVP, 6 cycles)
4	MiKail et al. [10]	58/female	Dilatation of IHD with a mass at liver hilus with intrahepatic biliary dilatation		4 cycles of R-CHOP (rituximab/cyclophosphamide/doxorubicine/vincristine/prednisolone)
5	This case	86/female	Diffuse dilatation of IHD and wall thickening of CHD	Intraluminal mass in the proximal CBD and on the duodenal side, a spreading tumor-like lesion	Radical bile duct resection + Roux-en-Y hepaticojejunostomy

*MALT* mucosa-associated lymphoid tissue, *IHD* intrahepatic bile duct, *EHD* extrahepatic bile duct, *CBD* common bile duct, *CVP* cyclophosphamide, vincristine, prednisolone, *CHOP* cyclophosphamide, doxorubicin, vincristine, and prednisolone, *CT* computed tomography, *ERCP* endoscopic retrograde cholangiopancreatography, *MRCP* magnetic resonance cholangiopancreatography, *MRI* magnetic resonance imaging, *PET* positron emission tomography

The three patients who had undergone surgery were males aged 59 to 71 years (mean 64 years). They had been admitted to the hospital for jaundice with mean total and direct bilirubin levels of 11.15 and 7.85 mg/dL. Radiological studies revealed that the perihilar bile ducts were diffusely thickened, with irregular, incomplete stenosis or long circumferential wall thickening shaped. All patients who underwent surgery had received a preoperative diagnosis of Klatskin tumor. On postoperative immunohistochemical studies, however, bile duct narrowing turned out instead to be related to MALT lymphoma. In three cases, partial hepatectomy combined with caudate lobectomy were also performed. In our case, a negative resection margin was secured without the need for further hepatic resection, as the proximal margin was preserved just below perihilar duct bifurcation.

To avoid misdiagnosing and inappropriately treating cases of MALT lymphoma resembling Klatskin tumor, several suggestions have been made. According to Shimura et al., evaluation of incomplete stenosis of the bile duct by 18-F fluoro-2-deoxyglucose positron emission tomography can distinguish MALT lymphoma from Klatskin tumor [7]. Yoon et al. propose that it is necessary to consider MALT lymphoma when cholangiography shows smooth luminal narrowing of the extrahepatic bile duct without mucosal irregularity, despite diffuse thickening of the ductal wall on CT and magnetic resonance imaging (MRI) [8]. Still, it remains difficult to diagnose primary MALT lymphoma in this region on either CT or MRI. MiKail et al. reported a unique case that was diagnosed without the need for surgical specimens [10]: a 58-year-old female patient was thought to have a Klatskin tumor and was prepared for major surgery; however, a type of lymphoma was suspected based on concerning biological behavior of the tumor (rapid growth resulting in doubling of size within a month), and the patient underwent a tru-cut biopsy via percutaneous transhepatic cholangiography (PTC), which led to the diagnosis of low-grade B cell lymphoma at the hepatic hilum without the need for surgery.

At the time of diagnosis, only 20–30 % of patients with Klatskin tumors are operable, leaving the remainder to follow a course of palliative treatment [11]. Biliary stents in both the right and left hepatic ducts help relieve jaundice and minimize the risk of cholangitis. With complications of stents, such as occlusion and migration, stent patency lasts only a few months before replacement becomes necessary. However, palliative stenting is actually inappropriate for patients diagnosed with primary MALT lymphoma mimicking Klatskin tumor. Whereas patients with inoperable Klatskin tumors can expect less than 1 year of survival, non-gastric MALT lymphomas have an indolent course, resulting in a 5-year survival rate of 93 % [12]. For the latter, chemotherapy or resection of space-occupying lesions can improve patient quality of life and long-term survival.

The greatest number of patients with extrahepatic bile duct cancer lies within the 75–84 year age range at diagnosis [13]. Patients 70 years or older are referred to as the elderly, and advanced age has been considered to be a contraindication for surgery, out of fear that extended resection of hepatobiliary tumors is too risky. In addition, life expectancy for the elderly is usually underestimated [14]. In one study, 14 % of inoperable Klatskin tumors were due to advanced age and coexisting medical conditions [15]. The 86-year-old female patient in our case had previously been considered inoperable due to her advanced age but was in fact well on the way to the recovery after surgery. There has been a recent trend to change this preconception and allow elderly patients more opportunities to undergo surgery. Resection of hepatobiliary cancer can be offered to the elderly as well as to patients with few co-morbidities and good functional status [16]. A study of Veterans Administration (VA) patients aged 80 years and over showed that the 30-day mortality rate was better predicted by functional status than by chronological age [17]. If preoperative performance status is favorable, a forward-looking approach to major biliary surgery in elderly patients can provide them a chance for cure.

## Conclusions

Although primary MALT lymphoma in the perihilar bile duct is very rare, strong clinical suspicion can lead to an accurate preoperative diagnosis and subsequent cure by surgical resection, chemotherapy, or both.

## Abbreviations

CBD, common bile duct; CT, computed tomography; MALT, mucosa-associated lymphoid tissue; MRI, magnetic resonance imaging
